# Comparison of push-out bond strength and Si Q^n^ species distribution in blood-contaminated calcium silicate cement

**DOI:** 10.1038/s41598-026-58614-8

**Published:** 2026-06-22

**Authors:** En-Shi Jiang, Wonjoon Moon, Shin Hye Chung

**Affiliations:** 1 Department of Dental Biomaterials Science and Dental Research Institute, School of Dentistry, Seoul National University, 101 Daehak-ro, Jongno-gu, Seoul, 03080 Republic of Korea; 2 Department of Stomatology, the Affiliated hospital of Yanbian University(Yanbian Hospital), 133000 Yanji, Jilin P.R. China

**Keywords:** calcium silicate cement, blood contamination, push-out bond strength, Ca/Si ratio, ^29^Si MAS NMR, Health care, Materials science, Medical research

## Abstract

Calcium silicate-based cements (CSCs) play a pivotal role in endodontic applications due to their favorable properties. This study investigates how blood contamination, a common clinical challenge during endodontic procedures, affects the bonding performance and hydration of CSCs. Tooth specimens were filled with four CSC-containing products (ProRoot MTA: PM, Endocem MTA: EC, iRoot BP Plus: IB, and TheraCal LC: TC) and placed in either distilled water or blood for 7 days at 37 ℃. The push-out test and evaluations using an inverted microscope and scanning electron microscope (SEM) were performed to measure the bond strength and fracture mode. Energy-dispersive X-ray spectroscopy (EDS) was used to measure the elemental composition and the Ca/Si ratio. ^29^Si magic angle spinning nuclear magnetic resonance spectroscopy (^29^Si MAS NMR) analysis was conducted to investigate the structures of the calcium silicate, and the mean silicate length (MCL) was calculated. Blood contamination significantly reduced bond strength for PM and increased bond strength for IB; no significant changes were observed with EC or TC. Blood contamination increased the Ca/Si ratio of each specimen, which was associated with decreased hydration and MCL values. It also influenced cohesive, mixed, and adhesive fractures among different groups. Blood contamination impaired the mechanical reliability of most hydration-dependent CSCs due to disrupted silicate polymerization, with the exception of IB, which exhibited an anomalous increase in push-out bond strength. From a clinical standpoint, these findings highlight the importance of hemostasis before CSC placement and suggest that future CSC formulations should improve resistance to blood contamination to enhance patient care.

## Introduction

Calcium silicate-based cements (CSC) are dental materials that include mineral trioxide aggregate (MTA) and its variations^1–3^. MTA-type materials have been used in endodontic procedures, including canal filling, regenerative endodontics, direct pulp capping, partial pulpotomy, apexogenesis, apexification, and perforation repair^9,4–6^. However, the poor handling characteristics of the original ProRoot MTA and the need for mixing led to the development of pre-mixed paste types of CSC products^7,8^, which are easy to use clinically. They do not require mixing a powder with a liquid before use^9^. Newer products set faster than the original ProRoot MTA and do not discolor (no bismuth oxide) and exhibit good cytocompatibility^10,11^.

Limited understanding exists how blood contamination alters the bonding performance and hydration behavior of CSCs, which may compromise sealing ability and long-term clinical outcomes. X-ray powder diffraction (XRD) and Fourier transform infrared spectroscopy (FTIR) provide limited information about the hydration and structure of calcium silicate. The ^29^Si magic angle spinning nuclear magnetic resonance spectroscopy (^29^Si MAS NMR) technique has been employed to characterize silicate crystallization and hydration process^12–14^. Microstructural integrity of the silicate network dictates the long-term mechanical stability and sealing ability of the cement in blood-contaminated environments.

This study aims to determine the effect of blood on the clinical application of CSC by measuring the push-out bond strength, evaluating fracture patterns, assessing the Ca/Si ratio, discriminating among^29^Si Q^n^structural species, and calculating hydration and the mean silicate chain length.

## Materials and methods

### Specimen preparation

The four dental materials used in this study (ProRoot MTA: PM, Endocem MTA: EC, iRoot BP Plus: IB, and TheraCal LC: TC) are described in Table 1. The Institutional Review Board (IRB) of the School of Dentistry, Seoul National University (IRB No. S-D20200050) approved the use of sixty extracted human premolars and autologous blood from the corresponding author. Written informed consent was obtained from all tooth donors, and all experiments were conducted in accordance with relevant ethical guidelines. The extracted teeth were stored in 0.1% thymol (Sigma Aldrich, Saint Louis, MO, US) at 4 ℃ until use. Only premolars with a separate straight root were selected.

**Table 1:** Materials used in this study.

Base	Materials (Code)	Composition	Manufacturer (Lot number)
Calcium Silicate	ProRoot MTA (PM)	Tricalcium silicate Dicalcium silicate Tricalcium aluminate Tetracalcium aluminoferrite Gypsum Free calcium oxide Ismuth oxide Distilled water	Dentsply Tulsa Dental, Johnson City, TN, USA (00000264122)
Calcium Silicate	Endocem MTA (EC)	Tricalcium silicate Calcium aluminate Calcium sulfate Lithium carbonate Zirconium dioxide HPMC Bentonite DMSO Water Ethyl alcohol	Maruchi, Wonju, Gangwon, Korea (FD200527C)
Calcium Silicate	iRoot BP Plus (IB)	Tricalcium silicate Zirconium oxide Tantalum pentoxide Dicalcium silicate Calcium sulfate	Innovative BioCeramix, Vancouver, BC, Canada (2001BPPS)
Light curing resin-modified calcium silicate	TheraCal LC (TC)	Calcium oxide Sr glass Fumed silica Barium sulfate Barium zirconate Type III Portland cement Bis-GMA HEMA UDMA TEGDMA PEGDMA	Bisco, Schaumburg, IL, US (2100003037)

**Fig. 1:**
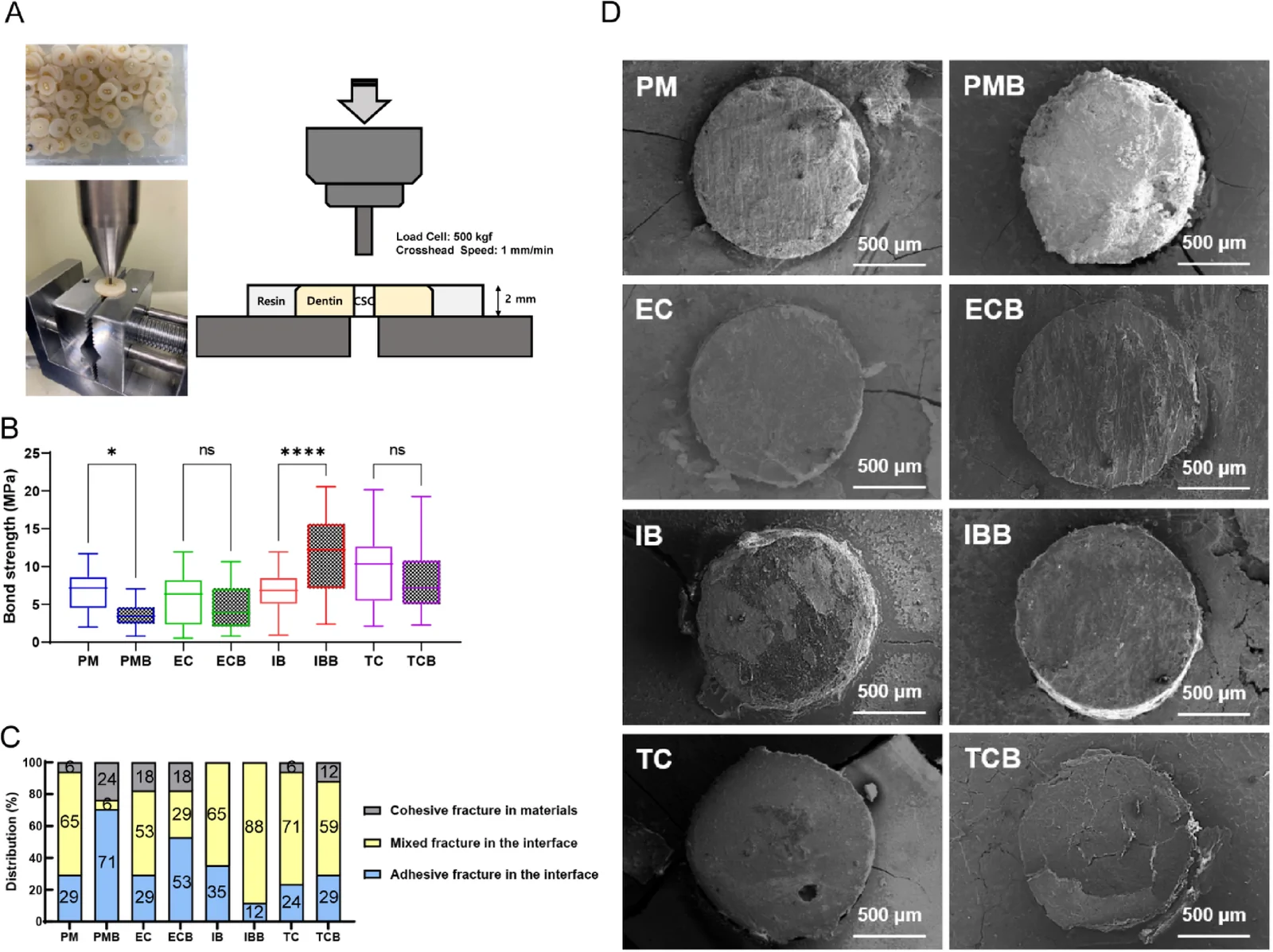
(**A**) Prepared samples and push-out test. (**B**) Push-out strength values across different groups (analyzed using Kruskal-Wallis and Bonferroni post hoc tests): *p < 0.05, ****p < 0.0001, ns: not significant. (**C**) Push-out fracture mode distribution in different groups classified into three types: cohesive fracture within materials, mixed fracture at the interface, and adhesive fracture at the interface. (**D**) Scanning electron micrographs (SEM, 200x) showing the vertical plan view of the flat top surfaces of the dislodged cylindrical CSC core disks after the push-out test. They were in contact with either DW or blood for 7 days at 37 ℃.

The crowns were removed at the cement-enamel junction, and the selected roots were separated with a diamond disc (Komet, Lemgo, Germany). The roots, approximately 9–10 mm in length, were cut into 2 mm sections using a low-speed saw (MECATOME T210, Presi, France). The root canals were prepared to a final diameter of approximately 1.0 ± 0.2 mm using a carbide round bur (FG2-010, Mani, Hanoi, Vietnam) (Fig. 1A). After preparation, the roots were irrigated with saline (NaCl, 0.9%, JW Pharmaceutical, Seoul, Korea) and dried with paper points (Diadent, Cheongju-si, Korea). The roots were randomly divided into four groups, 60 roots per group for the four materials. Each group was further divided into two subgroups of 30 each for exposure to blood or water.

The tooth slices were placed in distilled water (DW) or blood to moisten the interior of the root canal. PM was mixed according to the manufacturer’s protocol before filling; the other materials did not require mixing. All the canals were filled using a BL S-Kondenser (#35) (B&L Biotech, Ansan, Korea). TC was light-cured for 20 s using a 9 mm-diameter light-curing unit operating at a wavelength of 430–480 nm (Elipar DeepCure-S, 3 M ESPE, Seefeld, Germany). A wet cotton pellet was placed on the material. Then, each slice was placed on a membrane (Lyoplant, Aesculap AG, Tuttlingen, Germany) soaked with 100 µL of DW or blood so that the material was filled under moist or blood-contaminated conditions, rather than after complete setting. Fresh autologous blood was used to moisten both the root canal lumen and the supporting membrane, ensuring continuous contact between the unset cement and blood. The membrane-supported samples were positioned in the lids of inverted EP tubes, sealed, secured on the vial stands, and stored at 100% humidity at 37℃ for seven days before being used in subsequent experiments. TheraCal LC, a resin-modified and light-cured CSC, was included as a clinically relevant comparator to evaluate how different curing mechanisms influence material response to moisture and blood contamination.

### Push-out test

The push-out test to measure the bond strength of materials to dentin was performed by pushing the CSC from the top surface of each disc using a universal testing machine (UTM, TW-D102, Taewon Tech, Bucheon, Korea). The machine was equipped with a 500 kgF load cell and operated at a cross-head speed of 1.0 mm/min, as diagrammed in Fig. 1A, using a stainless-steel plunger with a diameter of 1.0 mm. The plunger was smaller than the prepared canal diameter and positioned to contact only the CSC surface without touching the surrounding dentin. The maximum force (N) recorded during dislodgement was divided by the bonded surface area (mm²) to calculate the bond strength (MPa). The bonded area was determined using the formula A = π × d × h, where d is the plunger diameter and h is the specimen height.

### Microscopic evaluation of fracture modes

The failure modes of each specimen were examined by two independent operators using a KS-208, Optinity stereoscopic microscope (Korea Lab Tech, Seongnam, Korea) equipped with an autofocus camera (KCX-50 F, Optinity) at 4x magnification. Three classifications, based on previous literature^15^, were used:^1^ AD: Adhesive failure between CSC and dentin;^2^ MX: Mixed (adhesive and cohesive) failure between CSC and dentin;^3^ CM: Cohesive failure within CSC.

### SEM-EDS analysis

For microstructural evaluation, the top surfaces of the cylindrical CSC core specimens, which had been dislodged from the root canals after the push-out bond strength test, were evaluated using scanning electron microscopy (SEM) (Apreo S, Thermo Scientific, Waltham, MA, USA) coupled with energy-dispersive spectroscopy (EDS). The samples were sputter-coated with platinum (sputter current: 20 mA, sputter time: 360 s. tooling factor: 2.3). SEM images were recorded under magnification of 200x (voltage: 10 kV, current: 0.1 nA, working distance: 10 mm). The elements were identified by EDS at a magnification of 1000x (voltage 20 kV, 0.4nA, working distance 10 mm), and the Ca-to-Si ratio was calculated.

### Analysis of ^1^H NMR and ^29^Si MAS NMR Spectroscopy

Spectra were obtained using a 500 MHz Avance III HD (Bruker, Bremen, Germany) spectrometer equipped with a 2.5 mm ^29^Si MAS NMR probe. The ^1^H NMR spectra were recorded with a 30-degree pulse of 1 µs, a pulse delay of 60 s, an acquisition time of ms, and a minimum of 16 scans. Solid-state ^29^Si NMR was used to distinguish among the various Si structures. Single-pulse ^29^Si MAS NMR spectra were obtained with a pulse delay of 60 s, an acquisition time of 50 ms, and 1000 scans, totaling 17 h. All spectra were collected with a spin rate of 10 kHz. In the ^29^Si MAS NMR spectra, the intensities of the various Q^n^ signals were plotted against a chemical shift scale referenced to tetramethylsilane (TMS). The raw data were processed using Topspin 3.5 software (Bruker) and Origin Pro software 2021 9.8.0 (OriginLab Corp., Northampton, MA, USA). The subtracted and deconvoluted spectra were then used to calculate the degree of hydration (HD), the mean silicate chain length (MCL), and the relative abundance of the various Q^n^ species according to the mathematical methods described previously^16–18^. Equations for calculating HD and MCL are as follows:

1 $$Degree\:of\:hydration\:\left(HD\right)=\frac{{Q}^{1}+{Q}^{2}+{Q}^{2B}+{Q}^{2L}+{Q}^{3}+{Q}^{4}}{{Q}^{0}+{Q}^{1}+{Q}^{2}+{Q}^{2B}+{{Q}^{2L}+Q}^{3}+{Q}^{4}}\times 100\%$$

2 $$Mean\:molecular\:chain\:length\:\left(MCL\right)=\frac{{Q}^{1}+{Q}^{2}+{Q}^{2B}+{{Q}^{2L}+Q}^{3}+{Q}^{4}}{\frac{1}{2}{Q}^{1}}$$

where Q^n^ refers to the silicate species-specific intensity of the ^29^Si MAS NMR peak. Q^0^ represents the uncondensed (unhydrated) silicate, Q^1^ is the disilicate/dimer (chain end group), and Q^2^ stands for the middle chain groups, within which Q^2B^ denotes bridging silica tetrahedra and Q^2L^ represents linear or paired chain silicates. The corresponding chemical shifts for these three species are approximately  -60 to -70 ppm, -71 to -76 ppm, and  -77 to -90 ppm, respectively^17,18^. The silicate tetrahedral structure of layers and chain-branching sites (Q^3^), and of three-dimensional networks (Q^4^), are included, corresponding to chemical shifts of approximately  -91 to 100 ppm and  -101 to 120 ppm, respectively^17^.

### Statistical analysis

The data were tested for normality by the Shapiro-Wilk test. Data were analyzed at a significance level of 0.05 using GraphPad Prism 9.0.0 (GraphPad Software Inc., San Diego, CA, USA). Statistical significance was assessed using the Kruskal-Wallis test and two-way analysis of variance (ANOVA), followed by a Bonferroni multiple comparison test.

## Results

### Push-out bond strength test

Push-out bond strengths are presented in Fig. 1A and B. Blood contamination significantly reduced the push-out bond strength of PM (*p* < 0.05), indicating compromised bonding under clinically contaminated conditions. The IB material bonded better with blood contamination (*p* < 0.0001). No significant differences were determined for the EC and the TC groups with or without blood contamination (*p* > 0.05). TC exhibited the significantly highest bond strength (*p* < 0.05) in water; the other materials were not significantly different from one another (*p* > 0.05). In blood, IBB had the significantly highest push-out strength, followed by TCB, ECB, and PMB (*p* < 0.01). No significant difference was determined between PMB and ECB groups (*p* > 0.05).

### Fracture mode analysis

Fracture modes are presented in Fig. 1C. Mixed fractures were observed in all groups, and cohesive fractures were also present in TCB. In both PM and TC groups, cohesive fractures increased after blood contamination, whereas EC showed no variation. No cohesive fractures occurred in the IB group, either with or without blood contamination. The PM, EC, and TC blood contamination groups had mixed fractures and more adhesive failures at the dentin–CSC interface, suggesting a higher clinical risk of interfacial debonding with inadequate hemostasis. Conversely, in the IB, blood contamination increased the mixed fractures and decreased adhesive fractures.

### Microscopic characteristics of CSC

SEM images depicting surface microstructural changes, are in Fig. 1D, showing a rough surface morphology for the two environments. No dramatic microstructural differences were observed; however, the blood-contaminated samples appeared slightly darker compared with those stored in distilled water, suggesting minor adsorption of blood components but not than deep penetration into the cement matrix.

### EDS analysis

**Fig. 2:**
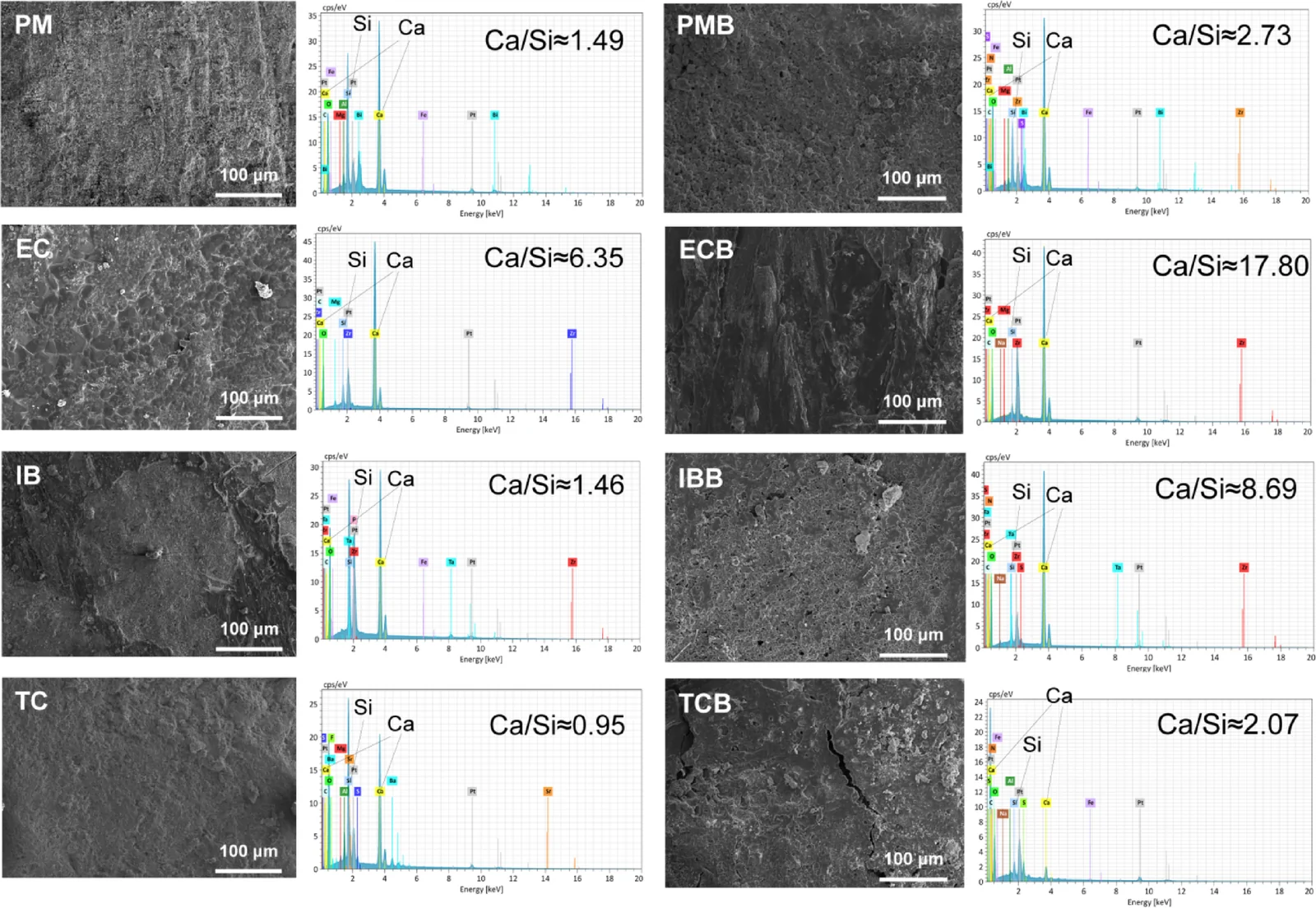
SEM images (1000x) and energy dispersive spectroscopy (EDS) spectra.

The EDS analyses demonstrated changes in the elemental composition of the materials in contact with different solutions (Fig. 2; Table 2). Peaks for Ca, O, and Si were observed in all CSC-containing materials, but the blood-contaminated groups showed a lower Si peak and a higher C peak, and a higher Ca/Si after 7 days. Alumina was detected in PM, PMB, TC, and TCB. Zr was observed in EC, ECB, IB, and IBB. Bi was detected in PM and PMB. Ta appeared in IB and IBB. Sr and Ba were identified in TC and TCB.

**Table 2:** EDS analysis of four types of CSC.

Elements	PM		PMB		EC		ECB		IB		IBB		TC		TCB	
	Mean	SD	Mean	SD	Mean	SD	Mean	SD	Mean	SD	Mean	SD	Mean	SD	Mean	SD
C	5.8	0.6	16.19	1.38	12.85	3.52	13.6	2.47	11.23	1.52	21.3	0.85	22.95	1.76	53.87	1.86
N			4.37	0.69			24.57	21.89			4.52	1.24			11.59	0.85
O	37.1	1.9	39.78	1.12	45.28	1.56	21.73	24.19	42.81	0.48	44.23	0.51	44.28	0.55	30.43	1.16
F													0.89	0.07		
Mg	0.59	0.05	1.08	0.19	1.19	0.73	0.79	0	0.67	0			0.73	0.13		
Al	1.75	0.13	0.99	0.07	0.06	0.1							2.48	0.1	0.57	0.18
Si	14.97	1.1	7.28	0.73	4.11	0.91	1.52	0.5	9.19	0.09	2.46	0.21	11.11	0.15	0.65	0.47
S	0.41	0.72	0.2	0.35	0.47	0.03			0.06	0	0.77	0.17	0.54	0.06	1.45	0.4
Ca	22.33	0.08	19.89	0.84	26.11	9.98	27.01	5.48	13.45	0.94	20.95	2.78	10.53	2.63	1.34	0.49
Fe	0.75	0.09	0.8	0.09	0.35	0.06	0.66	0.18	0.35	0	0.9	0			0.27	0.07
Zr			0.59	0.71	9.8	7	14.04	0.52	17.71	1.24	5.4	0.14				
Bi	16.29	3.57	8.81	0.94												
Ta									4.37	0.22	1.38	0.56				
P											0.45	0				
Sr													4.87	0.59	0.2	0
Ba													1.81	0.45	1.97	0.66

### Analysis of ^29^Si MAS NMR Spectra and ^1^H NMR spectroscopy

**Fig. 3:**
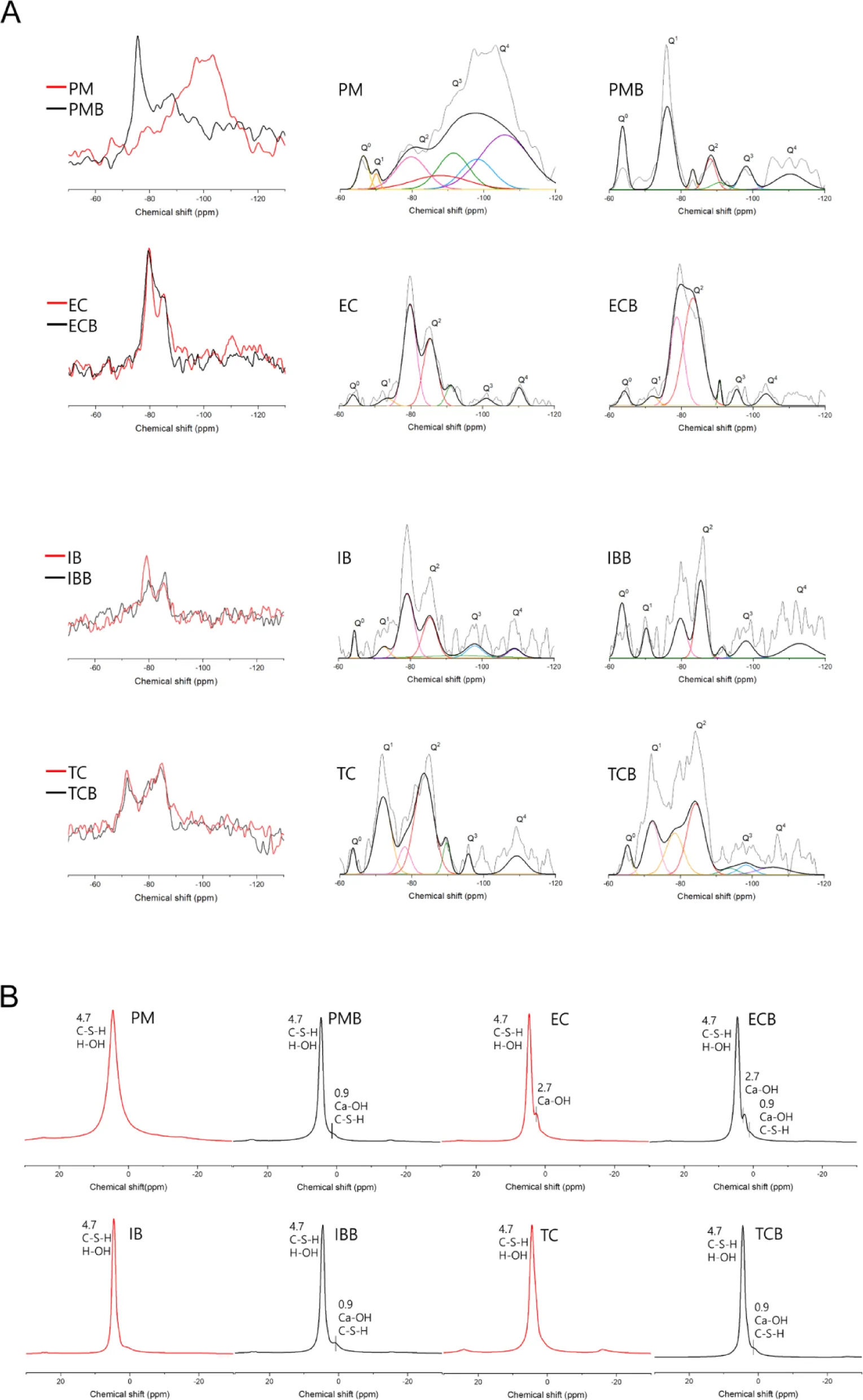
(**A**) The ^29^Si NMR spectra of the four materials, with and without blood contamination after 7 days. The ^29^Si NMR spectra are shown after subtraction, fitting, and deconvolution; water- and blood-contaminated materials. (**B**) The ^1^H NMR spectra of the four materials, with and without blood contamination after 7 days.

The ^2^^9^Si MAS NMR data were used to determine relative abundance of the Q^n^ species. The ^29^Si MAS NMR spectra displayed the visual appearance of Q^0^, Q^1^, Q^2^, Q^3^, and Q^4^, which are shown after overlapping and deconvoluting the signals in Fig. 3A. Significant changes in the peak intensities of Q^3^ and Q^4^ were noted in PM following blood contamination compared to the water-control group. The PMB spectra showed a high Q^1^ resonance peak intensity, indicating less hydration. EC and IB exhibited minimal variations in their respective Q^2^ peak intensities when comparing their water-control and blood-contaminated subgroups (EC vs. ECB, and IB vs. IBB). TC and TCB demonstrated very similar patterns in Q1 and Q^2^ peak intensities. Q^2^ peak intensities were higher in TCB compared to TC. The hydration and mean silicate chain length (MCL) of the four CSC-based products were higher in samples without blood contamination compared to those with blood contamination for all 4 materials (Table 3). ^1^H NMR spectroscopy results are presented in Fig. 3B. All four materials showed a prominent peak at 4.7 ppm, corresponding to ^1^H in C–S–H and H–OH. The blood-contaminated groups all displayed a 0.9 ppm ^1^H peak of Ca–OH from Portlandite (Ca(OH)_2_ (CH)) and C–S–H. A peak at 2.7 ppm in EC and ECB was identified as ^1^H in Ca–OH of α-dicalcium silicate hydrate, Ca_2_(SiO_3_)(OH)_2_^19^.

**Table 3:** Relative abundance of Q^n^ species, degree of hydration, and mean silicate chain length.

Sample	Q^0^ (%)	Q^1^ (%)	Q^2B^ (%)	Q^2^ (%)	Q^2L^(%)	Q^3^ (%)	Q^4^ (%)	Hydration (%)	MCL
PM	5.26	1.06	6.45	9.32	9.51	26.54	41.86	94.74	16.01
PMB	16.56	39.13	3.75	11.67	3.65	10.00	15.24	83.44	4.91
EC	3.08	3.61	45.00	31.94	7.24	3.31	5.82	96.92	52.57
ECB	3.17	2.76	32.09	53.34	1.98	3.08	3.59	96.83	51.47
IB	4.90	5.33	40.77	26.41	7.80	9.14	5.65	95.10	26.13
IBB	18.02	7.78	17.84	28.86	2.82	9.16	15.52	81.98	14.15
TC	3.39	28.02	7.66	44.59	5.40	2.99	7.94	96.61	22.16
TCB	6.68	21.88	21.23	36.26	2.99	4.33	6.63	93.32	7.65

## Discussion

Blood contamination is a frequent clinical challenge during endodontic procedures. This study demonstrates that blood exposure altered the Si Q^n^ species distribution, as evidenced by our NMR and EDS analyses.

Our solid-state NMR quantitative results (Table 3) directly explain the macro-mechanical bond strength behavior. The distinct reduction in MCL observed in blood-contaminated PM, EC, and TC directly correlates with their compromised push-out resistance, confirming that internal chemical network disruption limits macro-mechanical bonding capacity. Blood contamination disrupted the crystallization of these C_3_S monomers into mature silicate chains at 7 days, as evidenced by the reduced MCL values across all blood-contaminated materials. The lower MCL and HD observed in blood-contaminated samples suggest that blood interfered with the hydration and crystallization of calcium silicate, which reduced the interconnectedness of the C–S–H network, which is clinically undesirable for bonding at the dentin–cement interface. The lower MCL and HC may clinically manifest as a weaker interface with dentin, manifested in the lower push-out bond strength with blood contamination, except for IB. Although some blood-contaminated groups exhibited higher Q^1^–Q^2^ or reduced Q^3^ peak intensities, these changes reflect disrupted rather than enhanced hydration, as confirmed by reduced HD and MCL across all materials. All the materials had relatively high hydration levels of more than 80% regardless of blood contamination; therefore, blood exposure only partially inhibited cement hydration. A lower HD does not necessarily imply reduced portlandite formation; instead, blood contamination may alter the balance between C–S–H formation and portlandite precipitation or lead to heterogeneous distribution of hydration products. Such altered hydration pathways can result in a defective, less interconnected C–S–H network despite a high overall hydration percentage, thereby explaining the observed reductions in MCL, and bond strength. Well-developed Q^2^–Q^3^–dominant C–S–H networks are desirable, but blood contamination hindered their maturation, thereby compromising the interfacial bonding of most tested cements. However, IB exceptionally maintained a high push-out bond strength; this anomalous behavior suggests that despite internal silicate chain disruption, a formulation-specific surface interaction or a beneficial shift toward mixed fracture modes at the dentin interface effectively compensated for the macro-mechanical loss.

Relatively large intra-group variations in MCL values were observed specifically within the PMB and IBB groups (as indicated by the standard deviations in Table 3). Non-uniform hydration and heterogeneous crystallization may be exacerbated by blood contamination. Blood components can interfere with ion diffusion and C–S–H formation, resulting in microstructural inhomogeneity in the silicate chain polymerization within the set cement matrix, rather than a uniformly interconnected network. Such heterogeneity in MCL suggests that even within the same material, regions with insufficiently polymerized (hydrated) silicate structures may exist, potentially compromising bonding and strength. Blood contamination may reduce the microstructural homogeneity of hydration products and increase structural inconsistency, contributing to variability in bonding performance.

Mixed fracture reflects the simultaneous occurrence of adhesive failure at the dentin–cement interface and cohesive failure within the cement. Changes in the proportion of mixed fractures should be interpreted as shifts in the balance between interfacial adhesion and internal material cohesion rather than as a distinct fracture mechanism. Blood contamination significantly increased the PM adhesive fracture, consistent with previous studies^20–22^. Increased adhesive failures in PM, EC, and TC particularly under contamination, indicate weaker dentin bonding.

The blood pH and ionic composition were not analyzed, but may influence hydration and dissolution of CSCs. Calcium hydroxide (Ca(OH)₂) generated during hydration^23^ increases the pH and promotes C–S–H formation; however, Ca(OH)₂ does react with phosphate or carbonate ions in blood, forming HA or calcium carbonate. distilled water was used as a phosphate-free storage medium rather than PBS which leads to surface hydroxyapatite formation. the use of distilled water isolated the blood-induced effects on silicate hydration and polymerization without phosphate-related confounding reactions.

Minor elements detected by EDS included Mg in the IB and TC groups. In addition, trace amounts of Fe were consistently observed across most specimens. Regarding blood-derived elements such as Na and P, they were either detected in trace amounts following contamination (e.g., P in IBB) or served to partially mask and dilute the surface-enriched phases of the cement. This surface adsorption of organic components and inorganic ions during early hydration effectively accounts for the reduced detectability or altered signals of certain minor elements in the blood-contaminated samples. EDS is inherently surface-sensitive and semi-quantitative; small variations in surface chemistry or topography can result in disproportionate changes in low-intensity elemental signals. The observed variations in Mg and F signals are interpreted as a surface masking or dilution effect caused by the adsorbed blood constituents, rather than true elemental loss from the cement matrix. Because their levels were inconsistent across groups and did not correlate with changes in Ca/Si ratio, hydration, or mechanical behavior, their impact on the material properties in this study is considered minimal. SDS/IFU cross-checking further supported those major detected elements matched manufacturer-reported compositions.

The minor macroscopic color changes were observed in blood-contaminated specimens, confirm the absorption of blood. Faster setting may be beneficial. Blood contamination did not significantly reduce the bond strength in EC and TC, which may be related to their reported setting time of approximately 5 min, which is markedly faster than PM (1.3–4.6 h)^24,25^. Contamination occurred during placement rather than after full setting, which is clinically relevant.

TC cures via resin polymerization rather than water-mediated hydration, making it inherently less susceptible to dilution or ionic interference from blood. IB, despite being premixed, requires more than 2 h to set, during which its non-aqueous vehicle diffuses out and external moisture diffuses in to initiate hydration; in a blood environment, this diffusion process entraps blood proteins and macro-ions within the core, directly causing the severe disruptions in the degree of hydration and MCL observed in our ^29^Si MAS NMR data for the IBB group (Table 3).

EC contains dimethyl sulfoxide as an organic vehicle, which may facilitate ion mobility and contribute to its relatively rapid early setting behavior; however, its specific mechanistic role was not isolated in the present study. Notably, the EC groups exhibited exceptionally high MCL values in both environments, which indicates that this specific cement inherently forms a highly continuous and densely interconnected linear silicate chain framework during hydration. This robust macromolecular network structure within the matrix effectively minimized chemical disruption from blood components, allowing the material to maintain its structural integrity and macro-mechanical stability.

Only the IB material showed a significant increase in push-out strength with blood exposure.The favorable bonding behavior of IB under blood contamination is interpreted as a formulation-dependent response supported by the distinct fracture mode characteristics of IB. IB exhibited an increase in mixed fractures and a decrease in adhesive fractures, with no cohesive failure observed regardless of blood exposure, unlike the other materials which showed an increase in adhesive fractures under blood contamination. The compositional and setting characteristics of IB contribute to enhanced interfacial stability under blood-contamination. The NMR data supports this because IB maintained the same Q^2^ intensities and relatively high MCL values even in the presence of blood. Its chemical formulation preserved molecular-level polymerization, which is essential for maintaining a reliable bond to the root canal wall in challenging hemorrhagic conditions.

The compositional characteristics of the four CSCs influenced their behavior. PM, a conventional CSC-based product, mixed with water, having with a long setting time, was most affected by blood contamination. EC, with faster setting, showed minimal change. The bonding in the IB group was least affected by blood. TC is resin-based and light-cured, and exhibited little environmental sensitivity. These differences explain the varying degrees of hydration and mechanical responses among materials. Clinicians should be aware that even if a material appears to set visually, blood contamination can secretly compromise its internal silicate architecture, as evidenced by our NMR analysis. Therefore, achieving a mature (hydrated) silicate network (high MCL) through rigorous hemostasis is a structural necessity for ensuring the long-term success of the treatment. Calcium silicate cements continue to undergo complex crystallization and maturation over about 28 days, these findings reflect the material’s initial vulnerability to blood contamination rather than its ultimate properties.

In summary, blood contamination adversely affected the mechanical reliability of hydration-dependent CSCs by disrupting the silicate polymerization (hydration). The ^29^Si MAS NMR analysis identified that the reduction in mean silicate chain length (MCL) was a primary cause for the decreased bond strength and altered fracture behavior observed in three materials with blood-contaminated specimens. Resin-based, light-cured TheraCal LC and Endocem MTA exhibited greater bond strength due to their rapid-setting while other hydration-dependent CSCs exhibited a significant reduction in the mean silicate chain length (MCL) and a subsequent decrease in the density of the core hydration matrix under blood-contaminated conditions. These findings highlight that achieving adequate hemostasis is not merely a clinical preference but a necessity to ensure a well-developed, hydrated- silicate network for long-term clinical reliability. Future CSC formulations should prioritize maintaining molecular-level silicate chain polymerization and network interconnectedness even under clinically contaminated conditions.

## Data Availability

The datasets used and/or analysed during the current study available from the corresponding author on reasonable request.

## References

[1] Darvell, B. & Wu, R. MTA—An hydraulic silicate cement: Review update and setting reaction. *Dent. Mater.* **27**, 407–422 (2011). 21353694 10.1016/j.dental.2011.02.001

[2] Prati, C. & Gandolfi, M. G. Calcium silicate bioactive cements: Biological perspectives and clinical applications. *Dent. Mater.* **31**, 351–370 (2015). 25662204 10.1016/j.dental.2015.01.004

[3] Dawood, A. E., Parashos, P., Wong, R. H., Reynolds, E. C. & Manton, D. J. Calcium silicate-based cements: Composition, properties, and clinical applications. *J. Investig. Clin. Dent.* **8**, e12195 (2017). 10.1111/jicd.12195 26434562

[4] Maneepraug, W., Laovanitch Srisatjaluk, R. & Jantarat, J. The comparison of strip perforation repair using ProRoot MTA and calcium silicate-based sealer with cold hydraulic technique; bacterial leakage and micro-CT studies. *BMC Oral Health*. **25**, 1457 (2025). 41013426 10.1186/s12903-025-06840-3 PMC12465750

[5] Ajram, J., Khalil, I., Gergi, R. & Zogheib, C. Management of an immature necrotic permanent molar with apical periodontitis treated by regenerative endodontic protocol using calcium hydroxide and MM-MTA: A case report with two years follow up. *Dent. J.* **7**, 1 (2019). 10.3390/dj7010001 PMC6473881 30609673

[6] Asgary, S. & Ehsani, S. MTA resorption and periradicular healing in an open-apex incisor: A case report. *Saudi Dent. J.* **24**, 55–59 (2012). 23960529 10.1016/j.sdentj.2011.08.001 PMC3723285

[7] AlAnezi, A. Z., Zhu, Q., Wang, Y-H., Safavi, K. E. & Jiang, J. Effect of selected accelerants on setting time and biocompatibility of mineral trioxide aggregate (MTA). *Oral Surg. Oral Med. Oral Pathol. Oral Radiol. Endod*. **111**, 122–127 (2011). 21176827 10.1016/j.tripleo.2010.07.013

[8] Janini, A. C. P. et al. Biocompatibility analysis in subcutaneous tissue and physico-chemical analysis of pre-mixed calcium silicate–based sealers. *Clin. Oral Investig*. **27**, 2221–2234 (2023). 36977761 10.1007/s00784-023-04957-9

[9] Hatipoğlu, Ö., Varlı Tekingür, E. & Pertek Hatipoğlu, F. Comparative clinical success of direct pulp capping materials: A network meta-regression of randomized clinical trials. *J. Dent.* **162**, 106073 (2025). 40886932 10.1016/j.jdent.2025.106073

[10] Marques, R. B., Baroudi, K., Santos AFCd, Pontes, D. & Amaral, M. Tooth discoloration using calcium silicate-based cements for simulated revascularization in vitro. *Braz. Dent. J.* **32**, 53–58 (2021). 10.1590/0103-6440202103700 33914003

[11] Kim, M., Yang, W., Kim, H. & Ko, H. Comparison of the biological properties of ProRoot MTA, OrthoMTA, and Endocem MTA cements. *J. Endod*. **40**, 1649–1653 (2014). 25052144 10.1016/j.joen.2014.04.013

[12] Engelhardt, G. & Michel, D. *High-resolution solid-state NMR of silicates and zeolites* (U.S. Department of Energy Office of Scientific and Technical Information, 1987).

[13] Maekawa, H., Maekawa, T., Kawamura, K. & Yokokawa, T. The structural groups of alkali silicate glasses determined from 29Si MAS-NMR. *J. Non-Cryst. Solids*. **127**, 53–64 (1991).

[14] Cong, X. & Kirkpatrick, R. J. 29Si MAS NMR study of the structure of calcium silicate hydrate. *Adv. Cem. Based Mater.* **3**, 144–156 (1996).

[15] Pang, N-S., Jung, B-Y., Roh, B-D. & Shin, Y. Comparison of self-etching ceramic primer and conventional silanization to bond strength in cementation of fiber reinforced composite post. *Materials* **12**, 1585 (2019). 31096562 10.3390/ma12101585 PMC6567077

[16] Li, Q. & Coleman, N. J. The hydration chemistry of ProRoot MTA. *Dent. Mater. J.* **34**, 458–465 (2015). 26235710 10.4012/dmj.2014-309

[17] Kunther, W., Ferreiro, S. & Skibsted, J. Influence of the Ca/Si ratio on the compressive strength of cementitious calcium–silicate–hydrate binders. *J. Mater. Chem. A*. **5**, 17401–17412 (2017).

[18] Li, Q., Hurt, A. P. & Coleman, N. J. The application of 29Si NMR spectroscopy to the analysis of calcium silicate-based cement using Biodentine™ as an example. *J. Funct. Biomater.* **10**, 25 (2019). 31151191 10.3390/jfb10020025 PMC6617092

[19] Méducin, F., Bresson, B., Lequeux, N., De Noirfontaine, M. N. & Zanni, H. Calcium silicate hydrates investigated by solid-state high resolution 1H and 29Si nuclear magnetic resonance. *Cem. Concr. Res.* **37**, 631–638 (2007).

[20] Nekoofar, M. H., Stone, D. F. & Dummer, P. M. H. The effect of blood contamination on the compressive strength and surface microstructure of mineral trioxide aggregate. *Int. Endod. J.* **43**, 782–791 (2010). 20609024 10.1111/j.1365-2591.2010.01745.x

[21] Adl, A., Sadat Shojaee, N. & Pourhatami, N. Evaluation of the dislodgement resistance of a new pozzolan-based cement (EndoSeal MTA) compared to ProRoot MTA and Biodentine in the presence and absence of blood. *Scanning* **2019**, 3863069 (2019). 10.1155/2019/3863069 PMC6532292 31210836

[22] VanderWeele, R. A., Schwartz, S. A. & Beeson, T. J. Effect of blood contamination on retention characteristics of MTA when mixed with different liquids. *J. Endod*. **32**, 421–424 (2006). 16631840 10.1016/j.joen.2005.09.007

[23] Camilleri, J. Characterization and hydration kinetics of tricalcium silicate cement for use as a dental biomaterial. *Dent. Mater.* **27** (8), 836–844 (2011). 21600643 10.1016/j.dental.2011.04.010

[24] Che, J-L. et al. Comparison of setting time, compressive strength, solubility, and pH of four kinds of MTA. *Korean J. Dent. Mater.* **43**, 61–71 (2016).

[25] Ha, W. N., Nicholson, T., Kahler, B. & Walsh, L. J. Mineral trioxide aggregate—A review of properties and testing methodologies. *Materials* **10**(11), 1261 (2017). 29099082 10.3390/ma10111261 PMC5706208

